# Analysis of Whitefly Transcriptional Responses to *Beauveria bassiana* Infection Reveals New Insights into Insect-Fungus Interactions

**DOI:** 10.1371/journal.pone.0068185

**Published:** 2013-07-05

**Authors:** Jun Xia, Chang-Rong Zhang, Shan Zhang, Fang-Fang Li, Ming-Guang Feng, Xiao-Wei Wang, Shu-Sheng Liu

**Affiliations:** 1 Ministry of Agriculture Key Laboratory of Agricultural Entomology, Institute of Insect Sciences, Hangzhou, People's Republic of China; 2 College of Life Sciences, Zhejiang University, Hangzhou, China; Volcani Center, Israel

## Abstract

**Background:**

The fungal pathogen, *Beauveria bassiana*, is an efficient biocontrol agent against a variety of agricultural pests. A thorough understanding of the basic principles of insect-fungus interactions may enable the genetic modification of *Beauveria bassiana* to enhance its virulence. However, the molecular mechanism of insect response to *Beauveria bassiana* infection is poorly understood, let alone the identification of fungal virulent factors involved in pathogenesis.

**Methodology/Principal Findings:**

Here, next generation sequencing technology was applied to examine the expression of whitefly (*Bemisia tabaci*) genes in response to the infection of *Beauveria bassiana*. Results showed that, compared to control, 654 and 1,681genes were differentially expressed at 48 hours and 72 hours post-infected whiteflies, respectively. Functional and enrichment analyses indicated that the DNA damage stimulus response and drug metabolism were important anti-fungi strategies of the whitefly. Mitogen-activated protein kinase (MAPK) pathway was also likely involved in the whitefly defense responses. Furthermore, the notable suppression of general metabolism and ion transport genes observed in 72 hours post-infected *B. tabaci* might be manipulated by fungal secreted effectors. By mapping the sequencing tags to *B. bassiana* genome, we also identified a number of differentially expressed fungal genes between the early and late infection stages. These genes are generally associated with fungal cell wall synthesis and energy metabolism. The expression of fungal cell wall protein genes might play an important role in fungal pathogenesis and the dramatically up-regulated enzymes of carbon metabolism indicate the increasing usage of energy during the fungal infection.

**Conclusions/Significance:**

To our knowledge, this is the first report on the molecular mechanism of fungus-whitefly interactions. Our results provide a road map for future investigations on insect-pathogen interactions and genetically modifying the fungus to enhance its efficiency in whitefly control.

## Introduction

The whitefly *Bemisia tabaci* (Gennadius) (Hemiptera: Aleyrodidae) is a species complex that contains some of the most destructive pests of fiber, vegetable, and ornamental crops [1], [2], [3], [4] and causes severe economic losses every year by direct feeding, excreting honeydew and transmitting plant viruses [5], [6], [7], [8]. The whitefly is known to colonize over 600 plant species with high population growth rate and remarkable adaptability to environmental stresses [2], [9], making it rather difficult to manage *B. tabaci* and the virus it transmits. At present, more than 50 conventional insecticides have been employed to control the growth of *B. tabaci* populations and viral transmission [10]. Meanwhile, novel insecticides have also been developing for application [11], [12], [13]. However, utilizing chemical agents to control *B. tabaci* is facing ever-increasing difficulties due to the rapidly rising resistance to pesticides [14] and the overuse of insecticides further accelerates the resistance and causes severe harms to the environment.

The negative impacts of chemical pesticides encourage the development of alternative pest control strategies [10]. Among them, microbial control (especially with entomopathogenic fungi) is a great supplement to the conventional chemical control due to its effectiveness and environmentally friendly characteristics [15], [16], [17]. Currently, commercial products such as Mycotal® (*Verticillium lecanii*), Botanigard® (*Beauveria bassiana*) and PreFeRal® (*Paecilomyces fumosoroseus*) are available on the market for whitefly control [18]. In particular, *B. bassiana* has been proved to be an efficient and environmentally friendly biocontrol agent against a variety of pests [19], [20], [21], [22]. *Beauveria bassiana* infects insects by direct cuticle penetration rather than by ingestion or through a wound like viruses or bacteria [16]. The infection process consists of three stages: 1) attach to the cuticle, 2) penetrate the cuticle, and 3) proliferate in the haemocoel and kill the host. The whole process is rather complex and multiple host factors and fungal toxins could be involved in the process [23].

Although insect resistance to *B. bassiana* has not yet been reported, some disadvantages impede the wide use of fungal biological agents. For example, they are not as fast acting as chemical pesticides and their efficacy varies with field conditions. Successful application of *B. bassiana* needs favorable environmental conditions such as high humanity and medium temperature (not exceeding 32°C) [16]. These disadvantages limit the wide usage of *B. bassiana* [16]. In order to overcome these difficulties, it is critical to determine crucial virulent factors of *B. bassiana*, as well as to elucidate how insects respond to fungal infections [24], [25]. A thorough understanding of the basic principles of whitefly-fungus interactions may enable the genetic modification of the fungus to enhance its virulence to the whitefly as achieved in previous studies [26], [27]. However, the mechanism of *B. tabaci* response to the fungal infection is poorly understood, let alone the identification of virulent factors involved in fungal penetration into the whitefly cuticle.

The recently available whitefly transcriptome sequences [9], [28] in combination with the RNA-Seq technology, which is a revolutionary tool for measuring the levels of gene expression [29], [30], have provided us unprecedented opportunities to investigate the transcriptional response of *B. tabaci* to the fungal infection. Here, using the RNA-Seq technique, we identified differentially expressed genes in *B. tabaci* adults infected or not infected by *B. bassiana* and analyzed how the whitefly orchestrates its defense responses to the fungal infestation. We found that the mitogen-activated protein kinase (MAPK), DNA damage repair and drug metabolism related genes could be involved in the defense responses. We also noticed that the basal metabolism and several ion transporters were significantly down-regulated in the 72 hour post-infected whiteflies. Furthermore, by analyzing the differentially expressed *B. bassiana* genes, we identified several potential fungal virulence factors, which might be critical for *B. bassiana* to infect and modulate whiteflies. Altogether, our work provides the first report to reveal the molecular mechanisms of *B. tabaci* responses to the fungal infection and new insights into the fungus-whitefly interaction during the infection process.

## Materials and Methods

### Plants and Whitefly Cultures

The Mediterranean (MED) species of the whitefly *B. tabaci* species complex was used in all the experiments [2]. Whitefly cultures were maintained on cotton plants (*Gossypium hirsutum* L. cv. Zhemian 1793) in climate chambers at 27±1°C, 14 h of light and 10 h of darkness and 70±10% relative humidity. The insect cultures were monitored every 3–5 generations using RAPD-PCR (random amplified polymorphic DNA-polymerase chain reaction) with H16 primer to guarantee the population purity. The details for maintaining the whitefly stock cultures were described previously [7].

### Fungal Strain and Survival Curve Assay

The fungal strain *B. bassiana* ARSEF2860 from the RW Holley Center for Agriculture and Health (Ithaca, NY, USA) was preserved at 4°C on slants of Sabouraud dextrose agar. Conidia harvested from cultures grown for 7 days at 27°C and 95% relative humidity were suspended in 0.05% Tween 80 (10^10^ conidia/ml). Approximately 100 whitefly adults on a cotton leaf were placed in a Petri dish and incubated on ice for 30 s to make whiteflies stay still. Then the whitefly adults were exposed to a spray of 2 ml of the conidial suspension (10^10^ conidia/ml) in an Automatic Potter Spray Tower (Burkhard Scientific Ltd., UK) as described previously [31]. The sprayed whiteflies were reared *in situ* at the regime of 27°C and 95% RH and the number of living insects was recorded daily. Cadavers were moved to another moistened dish for incubation to verify whether they died of *B. bassiana* infection or not. For the control group, the same volume of 0.05% Tween 80 was sprayed onto the insects, followed by rearing under the same conditions. Each assay was repeated four times.

### Preparation of Whitefly Samples for Sequencing

Newly emerged adult whiteflies were collected into Petri dishes as described above. At first, control and 72 hours treatment groups were sprayed with 2 ml of 0.05% Tween 80 and conidial suspension, respectively. After 24 hours, the 48 hours treatment group was sprayed with 2 ml of conidial suspension. At 72 hours post-infection time point, approximately 1,000 living whiteflies were collected from the control, 48 hours and 72 hours treatments respectively. With this experimental design, all whiteflies were collected at the same developmental stage (72 hours post-emergence), which eliminates the effect of development on gene expression [32], [33]. These samples were immediately frozen in liquid nitrogen and then homogenated using the FastPrep system (MP Biomedicals). Total RNA was purified with SV total RNA isolation kit (Promega) according to the user’s guide. RNA purity and integrity were confirmed with Nanodrop 2000 (Thermo Scientific) and 2100 Bioanalyzer (Agilent) as previously described [34]. Three biological replicates for all of these samples were prepared separately, one sample was used for the sequencing library construction, and the other two was used for quantitative real-time PCR (qRT-PCR).

### Digital Gene Expression (DGE) Library Construction and Sequencing

For each sample, mRNA was purified from 6 µg total RNA with magnetic oligo (dT) and double strand cDNA were synthesized. The bead bound cDNA was subsequently digested with *Nla*III, which recognizes the CATG sites. Magnetic beads were used to purify the 3′ end cDNA fragments and Illumina adapter 1 was added to their 5′ ends. The purified cDNA fragments were then digested with *Mme*I, which cuts 17 bp downstream of the CATG site, to produce tags with adapter 1. Subsequently, Illumina adaptor 2 was added to the 3′ ends of the tags, acquiring 21 bp tags with adapter 1 and adaptor 2 at each end. After 15 cycles of PCR amplification, 6% TBE polyacrylamide gel electrophoresis was used to purify the tags. After digestion, single strand molecules were added to the Illumina sequencing flowcell and fixed. Each molecule was amplified *in situ* and the three samples were sequenced in parallel using Illumina HiSeq 2000 platform at Beijing Genomics Institute (Shenzhen, China). Each sample generated about 12 millions of 35 bp length reads.

### Tag Annotation and Data Normalization

Clean tags were generated after removing adaptor sequences, low quality sequences, empty reads and tags with a copy number of <2. A reference database containing all possible CATG+17 nucleotide tag sequences were created for the transcriptome of the MED species of the whitefly *B. tabaci* [9], [28]. Sequenced tags were mapped to the MED whitefly transcriptome reference database with no more than one nucleotide mismatch. Tags mapped to multiple reference sequences were filtered out, and the remaining tags were designated as unambiguous tags. The number of unambiguous tags for each gene was calculated for gene expression analysis and the number of transcripts per million tags (TPM) was used to normalize the data. As the extracted RNA and sequencing library may contain *B. bassiana* genes, we also mapped unambiguous tags to the *B. bassiana* genome [35] to examine the expression of *B. bassiana* genes. For gene expression analysis, the numbers of unambiguous tags for each *B. bassiana* gene were calculated and then normalized to TPM.

### Data Deposition

DGE library data sets obtained in this work are available at the NCBI Gene Expression Omnibus (GEO) under the accession number GSE38108.

### Analysis of Differential Expressed Genes

The levels of gene expression were compared between the control library and the 48 hours and 72 hours post-infection libraries, respectively. The differently expressed genes (DEGs) were determined using the standards of *p*<0.01, false discovery rate (FDR) <0.10 and the absolute value of log_2_ ratio ≥1 [36], [37]. The differently expressed *B. bassiana* genes were identified by comparing the 48 h and 72 h post-infection libraries. As no *B. bassiana* gene was present in the control library, it was excluded from comparison. Gene Ontology (GO) classification system was used to determine the possible functions of DEGs and the number of DEGs in each GO term was calculated. The MED transcriptome database was used as background to search for GO terms enriched with DEGs by hypergeometric test and p-value<0.05 was considered to be enriched. Similarly, all differentially expressed genes were mapped to the terms in the Kyoto Encyclopedia of Genes and Genomes (KEGG) database and the hypergeometric test was used to identify pathways significantly enriched with DEGs (p-value<0.05). To increase the reliability of our analyses, pathways or GO terms with less than 6 DEGs were filtered out. The *B. bassiana* secreted proteins were identified with SignalP3.0 (www.cbs.dtu.dk/services/SignalP/) by predicting the existence of signal peptides. Transmembrane domains were predicted using TMHMM Server 2.0 (www.cbs.dtu.dk/services/TMHMM/). Proteins predicted to contain a transmembrane domain in addition to the signal peptide were filtered out because these proteins likely to remain in the membrane upon secretion [38].

### qRT-PCR Analysis

To validate the results of DGE analyses, qRT-PCR experiments were performed on the ABI PRISM 7500 Fast Real-Time PCR System (Applied Biosystems) with SYBR-Green detection. To ensure the validity of the data, the sample in qRT-PCR analysis was from a biologically replicate independent from the DGE sequencing sample. For each gene, three replicates were analyzed and the average threshold cycle (Ct) was calculated. The relative expression levels were calculated with the 2^–ΔΔCt^ method [39]. Twelve whitefly genes and eleven fungal genes were selected to validate our DGE data. Similar procedure was used in the analysis of genes predicted encoding secretion proteins. To show the trend of expression level changes, the data at different time points were compared with that of 0 hpi. All designed primers were synthesized at Boshang BioCompany (Table S1). *Bemisia tabaci* β-actin was measured in parallel to normalize the expression levels of whitefly genes and *B. bassiana* 18S rRNA was used to normalize the expression levels of fungal genes.

## Results

### Whitefly Survival Curve

**Figure 1:**
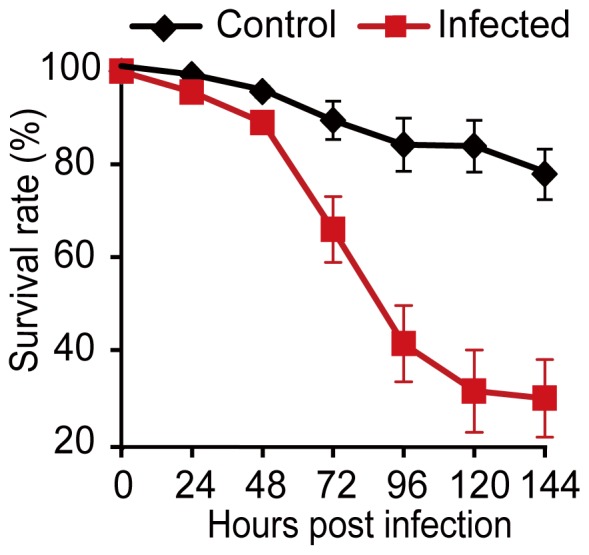
The survival curve of *B.* *bassiana* infected whitefly adults. Error bars: SE of the mean.

To verify the infectivity of fungal conidia and choose the most critical time points for comparison, the survival rate of the whitefly was monitored for 144 h after *B.* *bassiana* infection. As shown in Fig. 1, most of the whiteflies were alive after 48 h post infection (hpi), followed by a significant decrease at the time point of 72 hpi, suggesting 48–72 hpi is critical for the interaction between *B. bassiana* and *B. tabaci*. Since 72 hpi has been shown as an excellent time point for analyzing insect anti-fungal defense system in previous reports [32], [33], the time point of 72 hpi was chosen to investigate whitefly transcriptional responses. In addition, to reveal the earlier responses of *B. tabaci* to fungal infection, the time point of 48 hpi was also considered.

### DGE Library Construction, Sequencing and Mapping

The sequencing of three samples (∼1,000 whitefly adults per sample) from the control and the treatments of 48 and 72 hpi generated ∼12 million raw tags per sample. Saturation analysis showed the number of detected genes ceased to increase as the total tag number reached 10 million, which indicated our read number was enough (data not shown). Table S2 gives the basic parameters of the results from sequencing. The counts of distinct tags were 211,346, 217,260 and 215,923 in the libraries of control, 48 hpi and 72 hpi respectively (Table S2).

Since the copy number of a tag reflects the mRNA expression level, the distribution of tag expression was used to evaluate the normality of the DGE data [40] (Fig. S1). Among all three DGE libraries, similar tag distribution patterns over various abundance categories were observed, suggesting no bias for the constructed libraries. Most of the distinct tags appeared only a few times but a small percentage of them (5−6%) showed a frequency of more than 100 times in all three libraries (Fig. S1). This result was consistent with the normal mRNA distribution feature [9], [34], [40], [41], [42]. These distinct clean tags and their frequencies were deposited in the NCBI Gene Expression Omnibus (GEO) database with the accession number [GEO: GSE38108]. To reveal possible molecular events behind DGE profiles, the distinct tags were mapped to the available MED whitefly transcriptome reference database [9]. As a result, nearly 50% of them were mapped to ∼44,000 genes of the whitefly transcriptome, which also means about 25% of the genes in our reference transcriptome could be unambiguously identified by distinct tags (Table S2).

### Differentially Expressed Genes and qRT-PCR Validation

**Figure 2:**
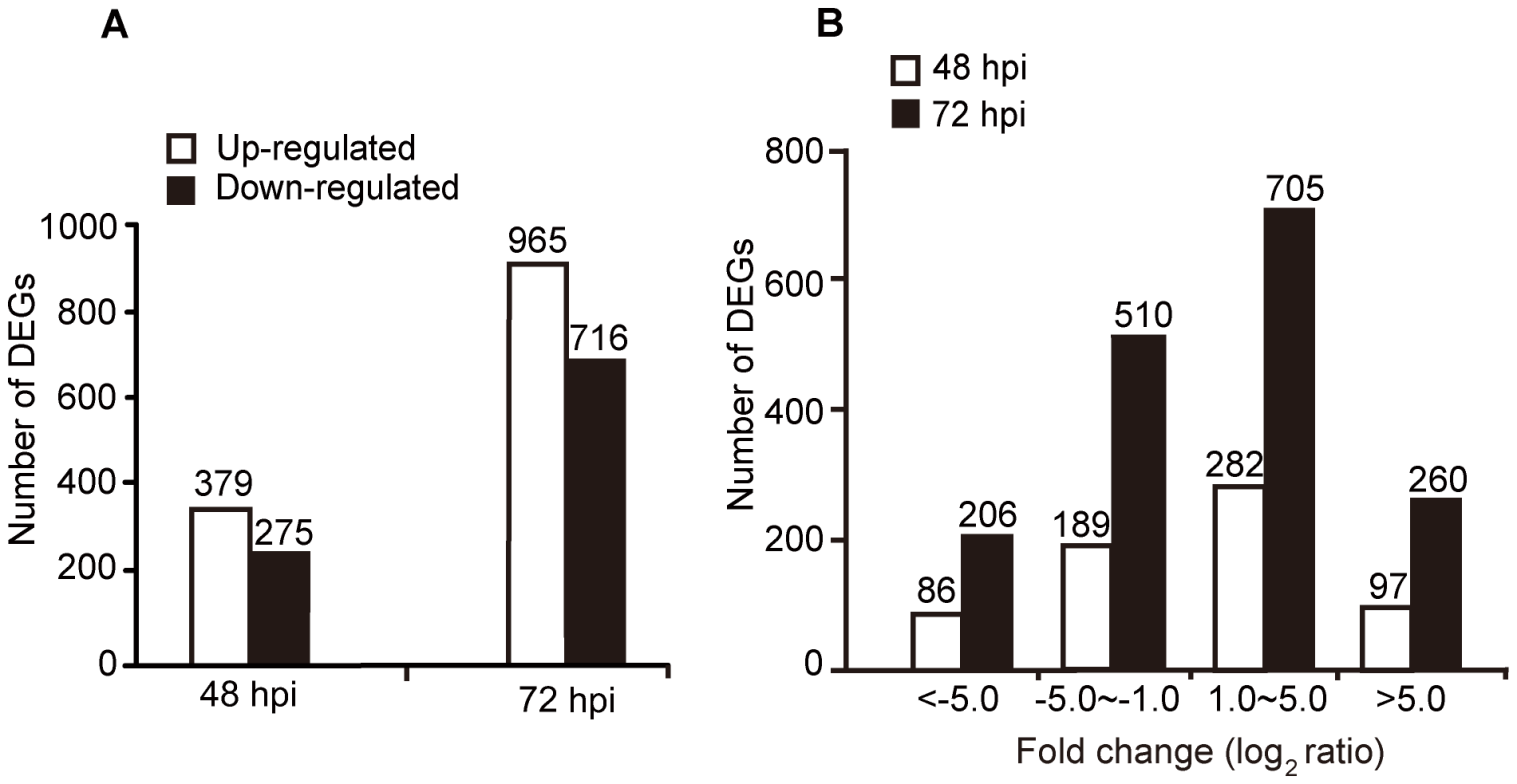
Analysis of differently expressed genes (DGEs). (A) An overview of DEGs between the whitefly libraries of 48 hpi and control, and of 72 hpi and control. The numbers of up- and down-regulated genes are marked above each bar. The white and black bars indicate the up- and down- regulated genes, respectively. (B) The distribution of fold changes (log_2_ ratio) of DEGs.

To identify DEGs, which may play vital roles in the defense responses of the whitefly to the fungal infection, two comparisons were performed: 1) control vs. 48 hpi; 2) control vs. 72 hpi. The analysis indicated 654 genes in control vs. 48 hpi, and 1,681 genes in control vs. 72 hpi exhibited significant changes (Fig. 2A and Table S3 in the supplemental material). The higher number of DEGs at 72 hpi was consistent with the bioassay results (Fig. 1) and further confirmed that 72 hpi was critical for *B. bassiana* action on the whitefly. Compared with the control, the 48 hpi treatment resulted in the up- and down-regulation of 379 and 275 genes, respectively. In the control vs. 72 hpi, 965 genes were up-regulated and 716 down-regulated (Fig. 2A). The fold change (log_2_ ratio) of the gene expression ranged from –7.72 to 8.43, and the majority of genes were up- or down-regulated within five folds (log_2_ ratio) (Fig. 2B, Table S3). Due to the relatively high cost of Illumina sequencing, only one sequencing run was performed for each sample. To validate the DGE data, 12 randomly selected genes were quantified for their transcript levels in qRT-PCR using RNA extracted from two biological independent replicates. In both of the two biological replicates, ten genes showed concordant changes between the DGE and qRT-PCR data (Table S1), suggesting that the DGE data are reliable. This is also in accordance with previous reports on the reliability of DGE analysis for the identification of DEGs [9], [34], [40], [41], [42].

### Gene Ontology (GO) Annotation and Enrichment Analysis

In order to further reveal the functions of the DEGs, GO annotation was conducted. In control vs. 48 hpi, 54 DEGs were mapped to GO biological process, 71 to molecular function, and 50 to cellular component. As for control vs. 72 hpi, 138 DEGs were identified to GO biological process, 166 genes to molecular function, and 122 genes to cellular component. Enrichment analysis helps to identify potential pathways and processes involved in specific biological functions or pathways [43], [44]. Therefore, the DEGs were clustered and the GO terms enriched with DEGs were identified through hypergeometric test [44]. We will mainly discuss the GO terms in the category of ‘Biological Process’ because it can reflect the biological processes directly. According to the criterion described above, 5 GO terms in the category of biological processes were significantly enriched with DEGs at 48 hpi and 9 GO terms were significantly enriched at 72 hpi (Table 1 and Table S4).

**Table 1:** Statistically enriched Gene Ontology terms in the category of ‘Biological process’.

GO ID	GO Term	Total genes^a^	DEGs^b^	p-value
**48 hpi**				
GO:0050877	neurological system process	164	6	0.01433
GO:0003008	system process	187	6	0.02737
GO:0044267	cellular protein metabolic process	725	17	0.03107
GO:0043412	macromolecule modification	530	13	0.03616
GO:0006464	protein modification process	486	12	0.03956
**72 hpi**				
GO:0006974	response to DNA damage stimulus	127	11	0.00264
GO:0006811	ion transport	102	9	0.00454
GO:0033554	cellular response to stress	176	12	0.01406
GO:0016071	mRNA metabolic process	90	7	0.01870
GO:0006281	DNA repair	75	6	0.02132
GO:0006412	translation	155	10	0.02989
GO:0006950	response to stress	309	17	0.03942
GO:0043170	macromolecule metabolic process	1602	70	0.04840
GO:0010467	gene expression	782	37	0.04936

a The number of genes mapped to each GO term in the whole MED transcriptome.

b The number of differentially expressed genes mapped to each GO term.

At 48 hpi, the GO terms ‘cellular protein metabolic process’ (GO:0044267), ‘macromolecule modification’ (GO:0043412) and ‘protein modification process’ (GO:0006464) were enriched with DEGs (Table 1). These results showed that the infection of *B. bassiana* activated the general macromolecule modification in host, though the exact functions of those processes remain unknown. In addition, the ‘neurological system process’ (GO:0050877) and ‘system process’ (GO:0003008) were also enriched at 48 hpi. In contrast, more GO terms with specific function were enriched at 72 hpi. The ‘response to stress’ and ‘cellular response-to-stress’ GO terms were enriched significantly which might be important in the defense of *B. tabaci* to the *B. bassiana* infection at 72 hpi. The most enriched GO term was ‘response to DNA damage stimulus’, further suggesting that the fungal infection may cause host DNA damage and thus trigger the whitefly defense responses. There were 11 DEGs involved in the host response to DNA damage (Table 2). Since the DNA damage caused by pathogen infection may trigger DNA repair response [45], [46] and host DNA repair proteins are directly involved in the regulation of gene expression in response to the pathogen infection [47], [48], the responsive genes found in the infected whiteflies could constitute a defense system against the *B. bassiana* infection. Beside stress response, the ‘Gene expression’, ‘translation’ and ‘mRNA metabolic process’ GO terms were also enriched, indicating the regulation of transcription and translation in anti-fungi response (Table 1).

**Table 2:** Table **2.** Genes involved in DNA damage response at 72 hpi^a^.

Gene ID	Homologous function^b^	Species	Accession no.	FC^c^
BT_Q_ZJU_Singletons33232	myosin heavy chain 95F, putative	*Pediculus humanus corporis*	XP_002429594.1	–1.5
BT_Q_ZJU_Singletons35129	PREDICTED: similar to cak1	*Nasonia vitripennis*	XP_001606664.1	–1.2
BT_Q_ZJU_Singletons34086	methyl-CpG binding domain protein 4	*Gallus gallus*	NP_990024.1	–5.9
BT_Q_ZJU_Cluster2290	Mediator of DNA damage checkpoint protein	*Acromyrmex echinatior*	EGI57450.1	5.9
BT_Q_ZJU_Singletons141436	DNA mismatch repair protein pms2	*Aedes aegypti*	XP_001660584.1	5.9
BT_Q_ZJU_Singletons35651	DNA repair protein RAD50	*Pediculus humanus corporis*	XP_002431806.1	–1.7
BT_Q_ZJU_Singletons146860	PREDICTED: DNA-directed RNA polymerases I, II,and III subunit	*Megachile rotundata*	XM_003708039	–1.6
BT_Q_ZJU_Singletons166990	serine/threonine-protein kinase grp-like	*Apis florea*	XM_003692122	1.2
BT_Q_ZJU_Singletons67699	DNA-directed RNA polymerase II 23 kDa	*Pediculus humanus corporis*	XP_002429002.1	–1.5
BT_Q_ZJU_Cluster2450	DNA repair endonuclease XPF	*Harpegnathos saltator*	EFN90056.1	5.9
BT_Q_ZJU_Singletons33690	BRCA1-A complex subunit MERIT40	*Acromyrmex echinatior*	EGI60318.1	–1.7

a The genes with fold change >2 fold (log_2_ ratio >1) and FDR <0.1 are considered to be significant.

b Homologous function: the function of the homologous gene.

c FC: fold change (log_2_ ratio) of gene expression, where ratio = TPM (72 hpi)/TPM (control).

### KEGG Pathway Enrichment Analysis

To investigate which pathway was significantly regulated during fungal infection, we also mapped DEGs to KEGG pathways. Among the 654 DEGs in control vs. 48 hpi and the 1,681 DEGs in control vs. 72 hpi, 63 and 150 genes were mapped to various KEGG pathways (Table S5). Enrichment analysis was subsequently conducted to identify the significantly influenced pathways (*p*<0.05). Between control and 48 hpi, 9 pathways were enriched and most of them were associated with carbohydrate metabolisms and drug metabolism (Table 3). Interestingly, all of the genes in drug metabolism including cytochrome P450, carboxylesterase (COE) and UDP-glucuronosyltransferase were up-regulated at 48 hpi (Table 4), strongly suggesting the activation of this pathway during the early response of whiteflies to the fungal infection. Regarding control and 72 hpi, we also observed the activation of several well-characterized enzymes in drug metabolism in 72 h post-infection whiteflies [49] (Table 4). Similar phenomena were observed in *Anopheles gambiae*, *Bombyx mori* and *Eurygaster integriceps* under microbial challenge [50], [51], [52]. We propose that the cytochrome P450, COEs and UDP-glucuronosyltransferases were essential components of whiteflies in the detoxification of toxin, such as cyclic peptide toxins, secreted by fungi [53], [54]. In addition, at 72 hpi, DEGs were significantly enriched in the Cell cycle, RNA transport, and metabolism pathways as well, which were absent in the 48 hpi (Table 3). These results indicated that the whitefly might activate additional defense strategies against the fungal infection at 72 hpi and this phenomenon was consistent with the infection process of *B. bassiana*.

**Table 3:** Statistically enriched KEGG pathway.

Pathway ID	Description		Total genes^a^		DEGs^b^		p-value		
**48 hpi**									
ko00500	Starch and sucrose metabolism		398		10		9.90E-05		
ko00040	Pentose and glucuronate interconversions		189		6		0.00083		
ko00140	Steroid hormone biosynthesis		227		6		0.00211		
ko00980	Metabolism of xenobiotics by cytochrome P450		235		6		0.00251		
ko00982	Drug metabolism - cytochrome P450		235		6		0.00251		
ko00983	Drug metabolism - other enzymes		321		7		0.00268		
ko00830	Retinol metabolism		244		6		0.00303		
ko04120	Ubiquitin mediated proteolysis		377		7		0.00645		
ko01100	Metabolic pathways		2921		25		0.02184		
**72 hpi**									
ko03013		RNA transport		381		15		0.00029	
ko00860		Porphyrin and chlorophyll metabolism		159		8		0.00176	
ko04110		Cell cycle		290		11		0.00254	
ko04111		Cell cycle - yeast		232		9		0.00534	
ko00500		Starch and sucrose metabolism		398		12		0.01001	
ko03015		mRNA surveillance pathway		201		7		0.02297	
ko00983		Drug metabolism - other enzymes		321		9		0.03667	
ko00140		Steroid hormone biosynthesis		227		7		0.04055	

a The number of genes mapped to each KEGG pathway in the whole transcriptome.

b The number of differentially expressed genes mapped to each KEGG pathway.

**Table 4:** Table **4.** Genes involved in drug metabolism.

Gene ID	Homologous function	Species	Accession no.	FC
**48 hpi**				
BT_Q_ZJU_Singletons18297	cytochrome P450	*Anopheles funestus*	ACG68818.1	6.21
BT_Q_ZJU_Singletons31848	COE2	*Bemisia tabaci*	ABV45411.1	2.28
BT_Q_ZJU_Singletons115366	UDP-glucuronosyltransferase 1–8 precursor, putative	*Pediculus corporis*	XP_002427365.1	1.91
BT_Q_ZJU_Singletons15128	UDP-glucuronosyltransferase 2C1-like	*Acyrthosiphon pisum*	XP_001943715.1	1.16
BT_Q_ZJU_Singletons145495	glucosyl/glucuronosyl transferases	*Aedes aegypti*	XP_001660381.1	1.12
BT_Q_ZJU_Singletons166670	similar to glucosyl/glucuronosyl transferases	*Tribolium castaneum*	XP_967606.2	1.12
BT_Q_ZJU_Singletons11249	Cytochrome P450 6k1	*Camponotus floridanus*	EFN71133.1	1.69
**72 hpi**				
BT_Q_ZJU_Singletons18297	cytochrome P450	*Anopheles funestus*	ACG68818.1	6.57
BT_Q_ZJU_Singletons25005	cytochrome P450 CYP6CX1v1	*Bemisia tabaci*	ACT68012.1	6.11
BT_Q_ZJU_Cluster481	COE2	*Bemisia tabaci*	ABV45411.1	5.90
BT_Q_ZJU_Singletons156492	COE2	*Bemisia tabaci*	ABV45411.1	2.64
BT_Q_ZJU_Singletons115366	UDP-glucuronosyltransferase 1–8 precursor, putative	*Pediculus humanus corporis*	XP_002427365.1	1.81
BT_Q_ZJU_Singletons6458	esterase FE4-like isoform 1	*Acyrthosiphon pisum*	XP_001951107.1	-1.10
BT_Q_ZJU_Singletons19646	similar to glucosyl/glucuronosyl transferases	*Tribolium castaneum*	XP_967924.1	-1.31
BT_Q_ZJU_Singletons16804	PREDICTED: similar to glucosyl/glucuronosyl transferases	*Tribolium castaneum*	XP_967606.2	-5.88
BT_Q_ZJU_Singletons25322	UDP-glucuronosyltransferase 2B16-like	*Oryctolagus cuniculus*	XP_002717180.1	-6.07

### Down-regulation of Metabolism and Ion Transportation in Late Infection Stage

**Figure 3:**
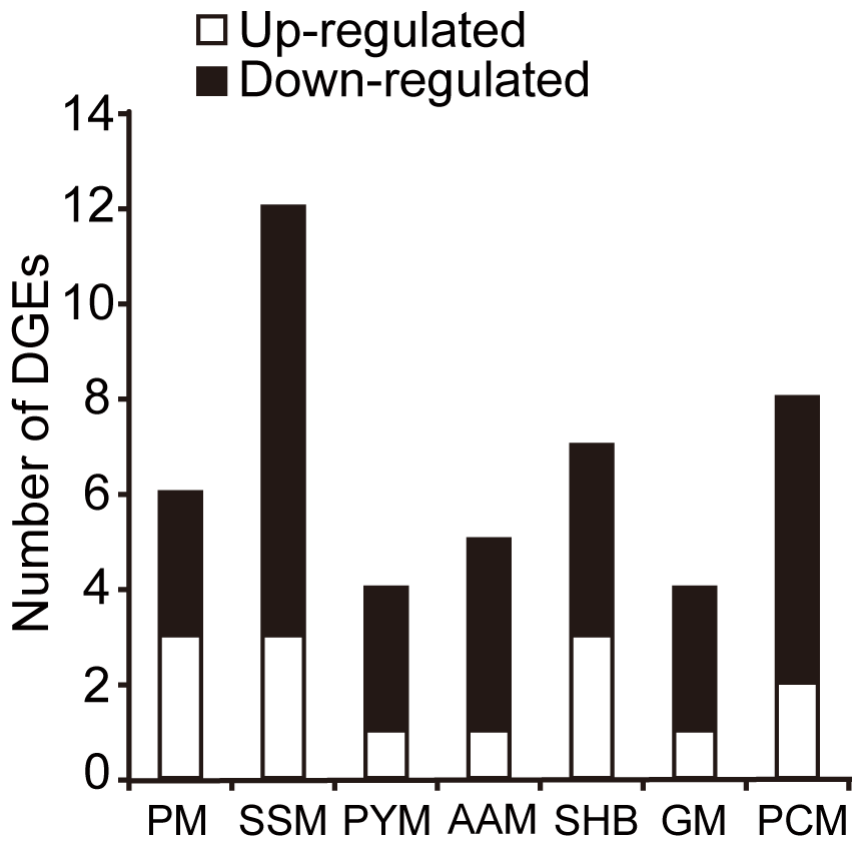
Expression of metabolism-related genes between whiteflies of 72 hpi and control. The numbers of up- and down- regulated genes are shown in white and black, respectively. The listed metabolism pathways are: purine metabolism (PM), starch and sucrose metabolism (SSM), pyrimidine metabolism (PYM), ascorbate and aldarate metabolism (AAM), steroid hormone biosynthesis (SHB), galactose metabolism (GM), porphyrin and chlorophyll metabolism (PCM).

In the bio-assay, the 72 hpi was featured by significant decrease of live whiteflies. Since we only collected live whitefly in our experiment, the 72 hpi data might present a picture of host in the late infection stage. In the 72 hpi treatment, nearly 70% of the genes involved in metabolism were down-regulated, including starch and sucrose metabolism, galactose metabolism, porphyrin and chlorophyll metabolism and steroid hormone biosynthesis (Fig. 3, Table S6). The same treatment also resulted in the down-regulation of 7 out 9 genes with GO term ‘ion transport’ (Table S6). Ion homeostasis, especially homeostasis of sodium, potassium and calcium is critical for multiple cellular functions [55], [56]. Moreover, three genes encoding ATP-binding cassette (ABC) transporters, which are known to involve in ion balance, immune signaling and resistance to fungal infection [57], [58], were also down-regulation at 72 hpi (Table S3). The down-regulated ion transporter and ABC transporter genes in the infected whiteflies could disorder the host immune response, as was evident with the disturbance of homostatsis by fungal virulent factors [59].

### Genes Involved in Immunity Signal Transduction Pathways

Because the transcriptome data we used only accounts for a part of the whole genome and lacks functional annotation, majority of the DEGs could not be annotated. Therefore, the GO and KEGG pathway analysis might not be able to present the whole picture of host defenses. To further explore the interactions between *B. tabaci* and *B. bassiana*, we also analyzed DEGs involved in immune signal transduction pathways. MAPK pathways are conserved in insect immune defenses and the defects of critical components of these pathways lead to host sensitivity to pathogen infection [32], [60]. In this study, three genes in the MAPK pathways were up-regulated (Table 5) at the time point of 72 hpi. In addition, a well characterized adaptor of Toll-like receptor - MyD88, which plays important roles in anti-fungal and anti-bacterial immune responses from human to insects [61], [62], was also up-regulated significantly in the 72 hpi treatment. These findings indicated that MAPK immune signal transduction pathway could be activated in the whitefly response to the fungal infection. The gene expression profile also showed that the two JAK-STAT pathway regulated genes, protein tyrosine phosphatase (PTP) and peroxidasin, were differentially expressed at the time of 72 hpi (Table 5), an indication for the involvement of the JAK-STAT pathway in the host anti-fungal defense [63]. In addition, several genes related to complement and coagulation cascades and melanization were suppressed at 72 hpi (Table 5). Interestingly, these genes were not differentially expressed at 48 hpi. Whether the fungal infection inhibits whitefly coagulation and melanization pathways warrants further investigation.

**Table 5:** Table **5.** Genes involved in the immune and defense related pathways.

Gene ID	Homologous function	Species	Accession no.	FC
**48 hpi**				
*** MAPK***				
BT_Q_ZJU_Singletons20021	Myeloid differentiation primary response protein MyD88	*Camponotus floridanus*	EFN62977.1	6.4
**72 hpi**				
*** MAPK***				
BT_Q_ZJU_Cluster2677	Nucleoprotein TPR	*Harpegnathos saltator*	EFN88236.1	2.7
BT_Q_ZJU_Singletons162105	putative calcium-binding protein p22	*Maconellicoccus hirsutus*	ABM55600.1	1.6
BT_Q_ZJU_Singletons31677	similar to protein tyrosine phosphatase, non-receptor type 11	*Tribolium castaneum*	XP_971440.2	1.1
***JAK STAT***				
BT_Q_ZJU_Singletons17253	peroxidasin-like	*Acyrthosiphon pisum*	XP_001948948.1	-2.1
BT_Q_ZJU_Singletons31677	protein tyrosine phosphatase, non-receptor type 11	*Tribolium castaneum*	XP_971440.2	1.1
*** Complement and coagulation cascades***				
BT_Q_ZJU_Singletons10195	elegaxobin-2	*Culex quinquefasciatus*	XP_001868417.1	-5.8
*** Melanoma***				
BT_Q_ZJU_Singletons166338	transcription factor E2F2-like isoform	*Acyrthosiphon pisum*	XP_001945228.1	-3.3
*** Melanogenesis***				
BT_Q_ZJU_Singletons31416	frizzled-2	*Culex quinquefasciatus*	XP_001868222.1	-6.1
BT_Q_ZJU_Singletons4970	calmodulin-A, putative	*Pediculus humanus corporis*	XP_002430223.1	-5.8

### Fungal Virulent Factors Involved in Whitefly-pathogen Interactions

**Figure 4:**
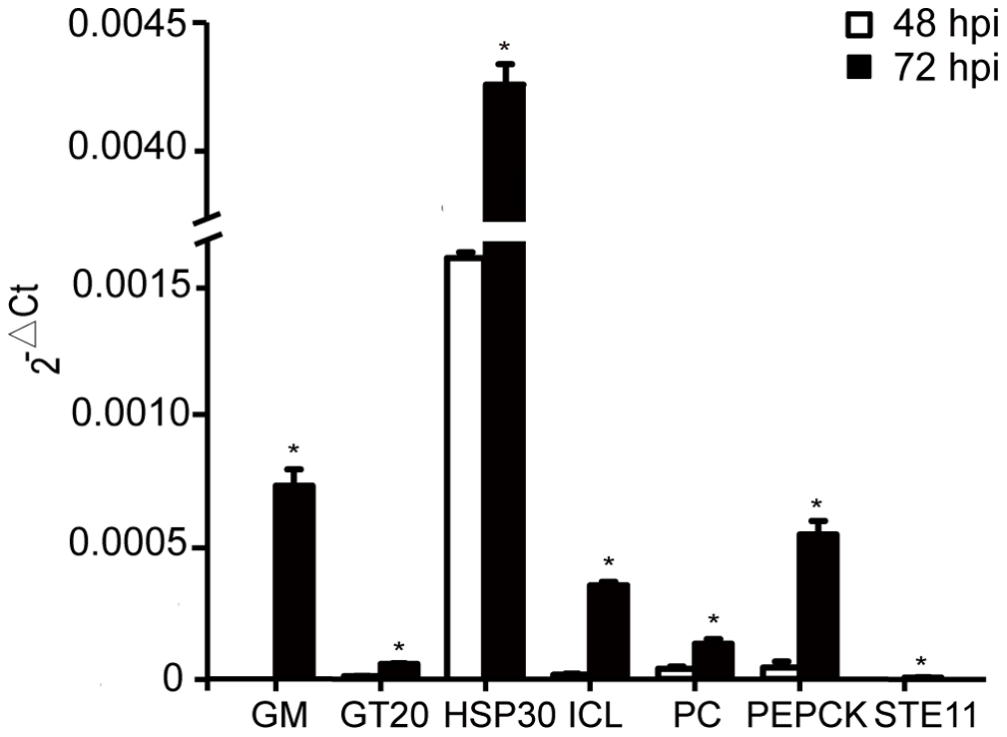
The expression levels (2^–Δ^ ^*Ctt*^ means) of selected fungal genes in whitefly adults infected by *B. bassiana* at the time points of 48 hpi (white) and 72 hpi (black). The assessed genes were galactomannoprotein (GM) (ID: BBA_05808), glycosyltransferase family 20 (GT20) (ID: BBA_08495), heat shock protein 30 (HSP30) (ID: BBA_02057), isocitrate lyase (ICL) (ID: BBA_01125), pyruvate carboxylase (PC) (ID: BBA_04309), phosphoenolpyruvate carboxykinase (PEPCK) (ID: BBA_02833) and MAPKKK Ste11 (STE11) (ID: BBA_02280), respectively. Error bars: SE of the mean.

To reveal the profiles of *B. bassiana* gene expression during infection, the DGE data were mapped to the sequenced genome of *B. bassiana* [35]. Because the majority of the sample for RNA extraction was the whitefly, only a small portion of the DGE tags (less than 0.01%) from the 48 and 72 hpi libraries were perfectly mapped to *B. bassiana* genes (Table S7). This strongly indicated that those *B. bassiana* genes were expressed during the infection. Comparing the expression levels of those genes between 48 and 72 hpi revealed that a number of fungal genes were up-regulated at the time of 72 hpi (Fig. 4), highlighting their vital roles in the process of the fungal infection. To confirm the sequencing data, the expression level of 7 selected *B. bassiana* genes at 48 hpi and 72 hpi were quantified using qRT-PCR. The experiment further confirmed that the candidate genes were significantly up-regulated at the time point of 72 hpi than 48 hpi (Fig. 4).

### Modulation of Cell Wall Synthesis and Regulation Related Genes

Functional analysis revealed that several differentially expressed *B. bassiana* genes were in association with cell wall synthesis and regulation. Of those, galactomannoprotein (BBA_05808) and glycosyltransferase (BBA_08495) genes were highly up-regulated at the time point of 72 hpi, as validated by qRT-PCR (Fig. 4). Polysaccharides and glycoproteins, such as cell wall galactomannoprotein, form antigenic surface layers that are important for regulating host response to fungal infection [64]. Furthermore, since glycosyltransferases are required for the synthesis of cell wall components, the highly up-regulated glycotranferase genes indicated their involvements in the fungal cell wall synthesis. This is in agreement with the recognized roles of these genes in the infection and virulence of other pathogens [65]. In addition, the MAPKKK-coding gene *ste11* (BBA_02280) possibly involved in regulating cell wall synthesis [66] was also up-regulated significantly at the time point of 72 hpi according to qRT-PCR analysis. Since *ste11* is known to regulate the downstream conserved MAPK pathway, the cell wall construction and growth in the yeast *Candida albicans* [66], it could also be involved in the construction and regulation of *B. bassiana* cell wall synthesis.

### Fungal Metabolism

As the host inner environment is rather complex, *B. bassiana* may adjust its metabolism to adapt to the host environment for successful infection. Interestingly, qRT-PCR analysis demonstrated that, as critical carbon metabolism enzymes, phosphoenolpyruvate carboxykinase (BBA_02833), isocitrate lyase (BBA_01125) and pyruvate carboxylase (BBA_04309) were expressed at the up-regulated levels of 12.1, 22.4 and 3.41 folds at 72 hpi compared to 48 hpi, respectively. These enzymes have been recognized as vital components of several biochemical reactions in other fungi and bacteria and deletion of these genes will cause significant defects of fungal virulence [67], [68], [69], [70]. As an acid-induced virulent factor [68], phosphoenolpyruvate carboxykinase together with pyruvate carboxylase is likely involved in the glucose metabolic process [70]. Isocitrate lyase is responsible for the glyoxylate cycle which may allow *B. bassiana* to utilize a variety of carbon sources during the insect pathogenesis [67], [68], [69], [71], [72]. The 22.4 fold up-regulation of isocitrate lyase probably indicated its very important role in the infection process. The elevated expression of the main enzymes associated with carbon metabolism indicated a high usage of energy and substances in the process of insect infection and also correlated to the up-regulation of HSP30 (BBA_02057) that may help *B. bassiana* to maintain fungal homeostasis and adapt to dynamic and challenging micro-environment in response to unconventional energy demand [73].

### Putative Secretion Proteins

**Figure 5:**
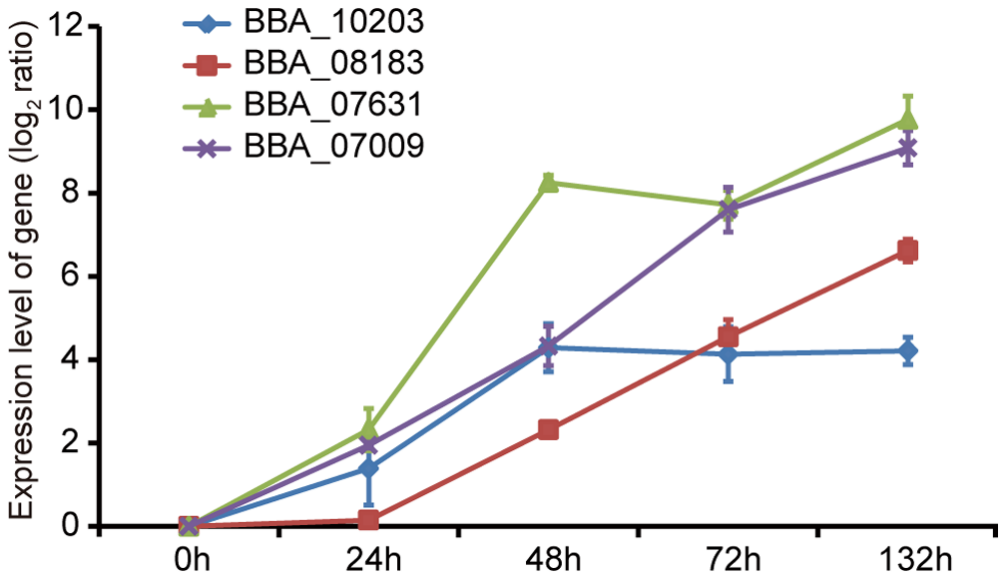
The relative expression level of four fungal genes encoding secretion proteins. The expression level of genes at the 24 hpi, 48 hpi, 72 hpi, 132 hpi were compared with that of 0 hpi and subjected to logarithm operation (log_2_ratio). The four genes BBA _10203, BBA_08183, BBA _07631, BBA _07009 were shown in blue, red, green and purple, respectively. Error bars: SE of the mean.

Interestingly, four of the differentially expressed fungal genes, BBA_10203, BBA_08183, BBA_07631 and BBA_07009, were found encoding potential secreted proteins (Table S7). Since the secreted proteins are probably crucial effectors involved in the fungal evasion and virulence [74], [75], the expression levels of those genes were determined at different infection stages. The data showed that the expression levels of all four genes were up-regulated remarkably, which was consistent with the DGE data (Fig. 5, Table S7). One of the genes, according to the blast result, BBA_08183 encodes a laccase which has been shown to be an important virulence factor of many pathogenic fungi [76]. In entomopathogenic fungus *Metarhizium anisopliae*, a laccase gene, Mlac1, was found to govern virulence and disrupting of Mlac1 resulted in impaired appressoria and delayed post-infection events [77]. The exact function of laccase in *B. bassiana* warrants further investigation.

## Discussion

*B. bassiana* is a promising fungal pathogen for *B. tabaci* control. The transcriptional analysis of *B. bassiana*-infected whiteflies helps to understand the interactions between *B. bassiana* and *B. tabaci*. Our study uncovers many pathways involved in whitefly defense responses to *B. bassiana* infection and at the same time, many interesting issues arise and need to be addressed. For example, it will be interesting to examine the exact roles of MAPKs in whitefly’s immune signaling. Our latest data showed the expression and phosphorylation of two MAPKs, JNK and p38, were activated in the *B. bassiana*-infected whiteflies (unpublished data). Further analyses are required to elucidate the function of MAPKs during fungal infection.

Even though a number of immune or defense pathways were identified in infected whiteflies, only a small proportion of the whitefly anti-fungal responses were revealed in our study. This is perhaps due to the following reasons. First, only the 48 hpi and 72 hpi whitefly samples were analyzed. Whiteflies probably utilize different immune and defense strategies in the earlier infection process. Second, the whitefly genome is not available yet and the transcriptome data used for annotation only accounts for a part of the whole genome, especially for the low expressed signal molecules. For example, many genes encoding immune response proteins, signal transduction molecules and antimicrobial peptides were absent in our reference database. For this reason, a large number of differentially expressed tags and genes could not be annotated and were missed in functional analysis. Third, *B. tabaci* might lack some of the immune genes found in *Drosophila* and use a different strategy to defend fungal infection, as revealed in pea aphid [78].

Moreover, in 72 h post-infected whiteflies, we observed an overall down-regulation of the general metabolism as well as ion transporter that may play important roles in the complex interactions between *B. tabaci* and *B. bassiana*. On the one hand, *B. tabaci* may reduce general metabolism and save energy to fight the fungal infection with alternative immune responses or reproductive strategies [79], [80], [81], [82]. On the other hand, this global down-regulation could be regulated by the fungal effectors that suppress the host genetic information processing and metabolism.

While there are reports of whitefly genes involved in insecticide resistance [83]; viral [84] and bacterial infections [85], few reports presented how entomopathogenic fungus regulates its gene expression during the infection of living insects. The few existing papers only examined the fungal response on specific *in vitro* tissues of insects rather than on live insects [24], [86], [87]. Since the fungus alters its gene expression pattern in different environment, previous studies may not reflect the real pattern of fungal gene expression during the infection of live insects. Our results showed that a number of potential virulence genes of *B. bassiana* were differentially expressed between the time points of 48 and 72 hpi. Galactomannanprotein seemed to play an important role in the fungal evasion, as revealed previously [64]. In *B. bassiana*-insect interactions, the absence of galactomannonprotein may allow the fungal cells to escape from the host immune detection and circulate freely in the host hemolymph [64]. In our study, no galactomannanprotein was expressed at the time of 48 hpi but its expression was dramatically induced at 72 hpi. This suggests a possibility that *B. bassiana* utilize this strategy to escape from recognition by the whitefly immune system. The highly up-regulated enzymes associated with carbon metabolism also indicate a large amount of energy consumed for successful fungal infection [67], [68], [69], [71], [72]. Also, special attention needs to be paid to the four putative secretion proteins and their roles in regulating host defense responses. Together with the genome sequence of *B. bassiana*, further investigation warrants a focus on the fungal genes involved in host infection and protein secretion. Our findings provide a valuable resource for future studies on the mechanisms of fungal infection and the genetic modification of fungal candidates for improved efficacy against *B. tabaci*.

In summary, we investigated the complex interactions between the whitefly and its fungal pathogen, *B. bassiana*, using NGS technology. Our data indicates that DNA damage stimulus response, drug metabolisms and MAPK pathways are likely involved in whitefly’s defense responses against *B. bassiana* infection. Furthermore, by analyzing the differentially expressed *B. bassiana* genes in different infection stages, we identified 108 fungal genes, which might be important for *B. bassiana* to infect and modulate whiteflies. To our knowledge, this is the first report about molecular interactions between *B. bassiana* and whitefly. Our results demonstrate complex interactions between *B. bassiana* and the whitefly, in which the insect has developed tactics to encounter the fungal infection, and *B. bassiana* has evolved strategies to ensure its colonization and pathogenicity.

## Supporting Information

Figure S1 **Distribution of distinct tags over different copy abundance in the three libraries (Control, 48 hpi and 72 hpi).** The digits in square brackets denote the copy numbers within a specific range. For example, two to five copies are expressed as [2], [5] in the tag category.
(PDF)

Table S1 **qRT-PCR primers and results.**
(XLS)

Table S2 **Overview of the DGE sequencing results.**
(DOCX)

Table S3 **List of differentially expressed genes.**
(XLS)

Table S4 **Results of Gene Ontology enrichment analysis.**
(XLS)

Table S5 **Results of KEGG pathway enrichment analysis.**
(XLS)

Table S6 **List of ion transport and metabolism related genes at 72 hpi.**
(XLS)

Table S7 **List of detected fungal genes.**
(XLS)
