# Supplementary figures and images for: Analysis of Whitefly Transcriptional Responses to Beauveria bassiana Infection Reveals New Insights into Insect-Fungus Interactions
Source: PLoS One. 2013 Jul 5;8(7):e68185. doi: 10.1371/journal.pone.0068185 (PMC3702578; doi:10.1371/journal.pone.0068185)

# Supplementary Figure 1

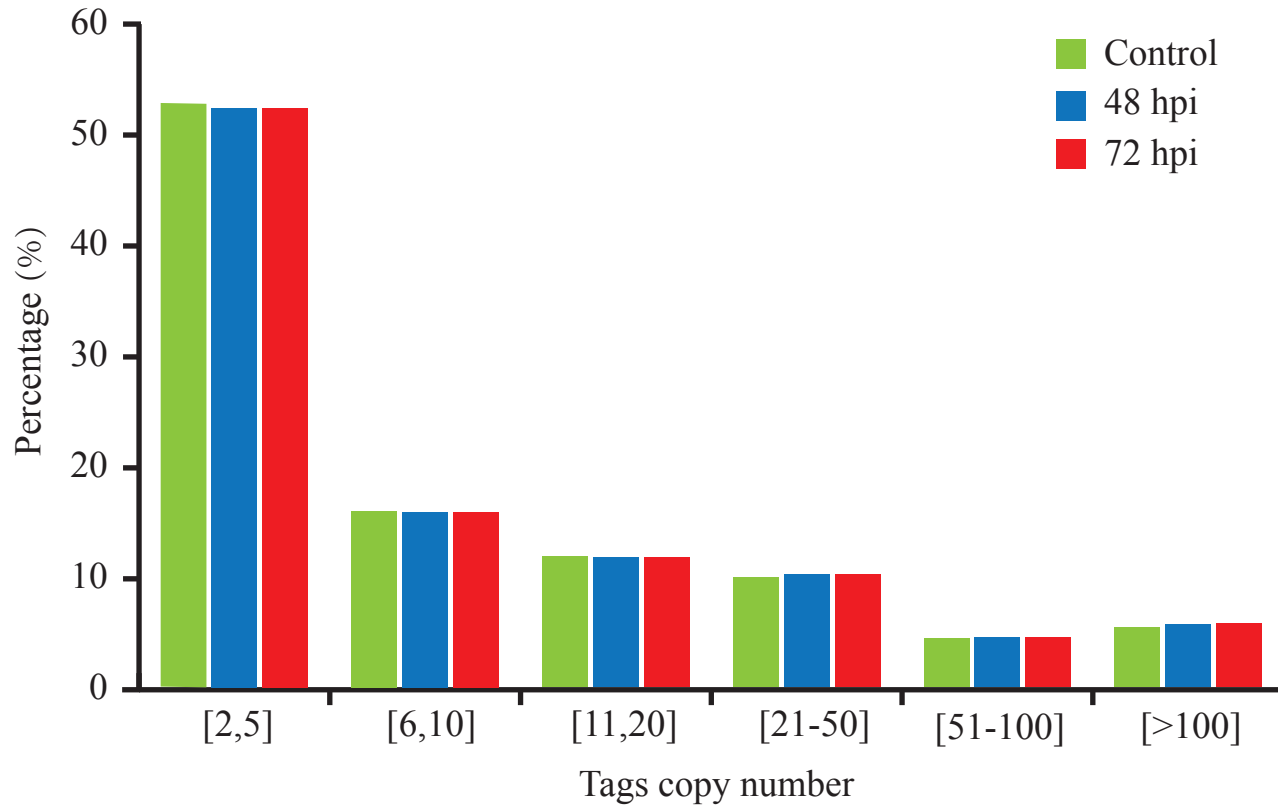

Supplement: Figure S1 — Distribution of distinct tags over different copy abundance in the three libraries (Control, 48 hpi and 72 hpi). The digits in square brackets denote the copy numbers within a specific range. For example, two to five copies are expressed as [2], [5] in the tag category. (PDF) [file pone.0068185.s001.pdf]
