# Supplementary material for: Analysis of Whitefly Transcriptional Responses to Beauveria bassiana Infection Reveals New Insights into Insect-Fungus Interactions
Source: PLoS One. 2013 Jul 5;8(7):e68185. doi: 10.1371/journal.pone.0068185 (PMC3702578; doi:10.1371/journal.pone.0068185)
Supplement: Table S2 — Overview of the DGE sequencing results. (DOCX) [file pone.0068185.s003.docx]

Supplementary Table 2**.** Overview of the DGE sequencing results

| Category | Parameter | Whitefly library | | |
| --- | --- | --- | --- | --- |
|  |  | Control | 48 hpi | 72 hpi |
| Clean tag | Total no. tags | 11869880 | 12233651 | 11571740 |
|  | No. distinct tags | 211346 | 217260 | 215923 |
| All tags mapping to genes | No. distinct tags | 107209 | 110523 | 109060 |
|  | % distinct tags | 50.73 | 50.87 | 50.51 |
| All tag-mapped genes | No. genes | 43295 | 44324 | 43919 |
|  | % referred genes | 25.63 | 26.24 | 26.00 |
| Unambiguous tag-mapped genes | No. genes | 42985 | 44009 | 43604 |
|  | % referred genes | 25.45 | 26.06 | 25.82 |
